# Neutrons & MnSOD: Past & Future

**DOI:** 10.1063/4.0000862

**Published:** 2025-10-27

**Authors:** Medhanjali Dasgupta, Gloria Borgstahl

**Affiliations:** University of Nebraska Medical Center, Omaha, NE, USA

## Abstract

Human manganese superoxide dismutase (MnSOD) is a tetrameric enzyme that plays a critical role in catalyzing the dismutation of superoxide radicals into molecular oxygen and hydrogen peroxide. This involves a redox cycle where the catalytic manganese alternates between oxidized (Mn^3+^) and reduced (Mn^2+^) states to facilitate coupled proton-electron transfer reactions (CPETs) that drive MnSOD function. The focus of the first half of this talk is to summarize the recent advancements in neutron protein crystallography and X-ray spectroscopy that have significantly enhanced our understanding of proton and electron transfer events within MnSOD, and revealed novel protonation states of key residues, such as Gln143 and Tyr34.

Additionally, the enzyme’s active site contains *unique hydrogen bonds,* including strong short hydrogen bonds (SSHBs) and low-barrier hydrogen bonds (LBHBs), which facilitate the enzyme’s *exceptionally* rapid catalytic turnover. Furthermore, product inhibition, driven by hydrogen peroxide accumulation, has been identified as a regulatory mechanism that shifts the enzyme’s catalytic pathway, leading to the formation of a “MnSOD product-inhibited complex”, which is resolved in the rate limiting step. Recent studies have characterized this complex, offering new insights into how MnSOD alleviates product inhibition. However, despite these advances, key questions remain regarding MnSOD's interaction with its *native* substrate, superoxide, and the precise structural mechanisms of substrate binding. The second half of the talk will focus on overcoming the current technological limitations and challenges to trapping the native substrate-bound MnSOD enzyme, which is essential to unraveling the full complexity of MnSOD catalysis.

